# Deep CNN-Based Static Modeling of Soft Robots Utilizing Absolute Nodal Coordinate Formulation

**DOI:** 10.3390/biomimetics8080611

**Published:** 2023-12-14

**Authors:** Haitham El-Hussieny, Ibrahim A. Hameed, Ayman A. Nada

**Affiliations:** 1 Department of Mechatronics and Robotics Engineering, Egypt-Japan University of Science and Technology (E-JUST), Alexandria 21934, Egypt; ayman.nada@ejust.edu.eg; 2 Department of ICT and Natural Sciences, Norwegian University of Science and Technology, 7034 Trondheim, Norway

**Keywords:** soft robots, continuum robots, statics modeling, deep learning, CNN

## Abstract

Soft continuum robots, inspired by the adaptability and agility of natural soft-bodied organisms like octopuses and elephant trunks, present a frontier in robotics research. However, exploiting their full potential necessitates precise modeling and control for specific motion and manipulation tasks. This study introduces an innovative approach using Deep Convolutional Neural Networks (CNN) for the inverse quasi-static modeling of these robots within the Absolute Nodal Coordinate Formulation (ANCF) framework. The ANCF effectively represents the complex non-linear behavior of soft continuum robots, while the CNN-based models are optimized for computational efficiency and precision. This combination is crucial for addressing the complex inverse statics problems associated with ANCF-modeled robots. Extensive numerical experiments were conducted to assess the performance of these Deep CNN-based models, demonstrating their suitability for real-time simulation and control in statics modeling. Additionally, this study includes a detailed cross-validation experiment to identify the most effective model architecture, taking into account factors such as the number of layers, activation functions, and unit configurations. The results highlight the significant benefits of integrating Deep CNN with ANCF models, paving the way for advanced statics modeling in soft continuum robotics.

## 1. Introduction

Recently, continuum robots have gained significant interest, as shown by the expanding research [1,2]. These robots have flexible backbones that allow for constant bending due to their elastic design, giving them notable dexterity, agility, and adaptability. Their capabilities make them ideal for tasks in tight spaces where flexibility, manevarability, and safe human interactions are crucial, as emphasized in newer research [3,4,5]. Unlike traditional rigid robots, continuum robots can change their forms dynamically, which makes them adept at moving through both tight and unpredictable spaces. This versatility makes them fitting for a range of uses, from inspecting industrial pipelines [6] and aircraft engine upkeep [7] to medical procedures [3,8].

Continuum robots are a specific subset of soft robots that are composed of a continuous, flexible structure that can bend and twist in any direction. They have a wide range of applications, including medical interventions [9,10], deep-sea explorations [11], and agricultural tasks [12,13].

Continuum robots utilize various actuation techniques, such as mechanical, fluidic, and magnetic methods. Mechanical actuation methods involve technologies like concentric tubes and backbone-driven actuators. Meanwhile, fluidic actuation covers designs like pleated, corrugated, belloscope tip-driven, and endoscope systems [14]. To unlock the full capabilities of continuum robots, accurate modeling and control are essential to performing specific movement and manipulation tasks [15]. However, their naturally flexible and malleable nature makes modeling their dynamics a complex task [16]. Unlike robots with rigid components, continuum robots present both distinct challenges and potentials in the creation of controllers [17,18]. One of the key challenges in controlling continuum robots is achieving precise tip control, which is necessary for performing delicate tasks in complex environments [19,20].

Various modeling techniques have been adopted, each varying in their base assumptions and scope of application. A thorough examination of these modeling strategies, their numerical methods, and an in-depth assessment of their practicality is presented in Armanini’s research [21]. One significant approach is the lumped mass model [22], where a flexible link is visualized as segments of point masses linked by springs and dampers. In this model, every mass is affected by gravitational forces and the movements of its neighboring masses, with some potentially facing external forces or actuator-induced interactions. The piecewise constant curvature model is another approach that has gained traction [23,24,25,26,27], simplifying robot kinematics by segmenting them into parameters that reflect constant-curvature segments. However, this method’s limitation lies in its strict adherence to constant curvature motions, occasionally neglecting crucial real-world influencers like gravity or friction.

Another model worth noting is the discrete elastic rod (DER) model introduced by Naughton et al. [28]. It is a computational approach used to simulate the behavior of thin, flexible rods in various applications, ranging from computer graphics to the study of biological fibers and soft robotics. Thus, a flexible rod is modeled using a series of lines connected at specific points and uses discrete differential geometry to capture bending and twisting. But, while advantageous in offering a balance between computational efficiency and physical accuracy, the DER model may not encapsulate the intricate designs seen in specific continuum robots. The simulation of the dynamics in soft material systems requires an intricate analysis of kinematics, mechanics, and tribology. These complexities might not be fully encompassed by the Discrete Elastic Rod (DER) model, which is generally employed for modeling slender structures such as rods, ribbons, and viscous threads [29].

Lately, finite-element-based models (FEM) have gained popularity in soft robotics [30,31,32]. These divide the robot’s structure into one, two, or three-dimensional elements, resembling the classification in conventional robotics. Beams and rods like the Euler–Bernoulli beam, Timoshenko beam, and the Cosserat rod provide the material deformation understanding of the robot [33]. However, despite their suitability for minor deformation challenges [34], FEMs come with their set of drawbacks such as computational overheads, potential for numerical instabilities, inaccuracies emerging from mesh alterations, and failing in the modeling of large deformations.

While the previously mentioned methodologies offer valuable insights into modeling continuum robots, there is a noticeable gap in exhaustive data-driven statics modeling and control techniques. This gap hinders the full exploitation of these robots across varied domains. Addressing this issue requires embracing data-driven modeling approaches, especially capitalizing on machine learning as underscored in a recent review by Wang et al. [35]. Given the swift progress in artificial intelligence areas, including machine learning, robotics, neural networks, transformers, and generative networks, incorporating data-driven statics modeling and control methods for soft continuum robots is promising. Such advancements can lead to significant benefits, such as improved accuracy, enhanced safety, and better operational efficiency in robotic applications [36].

In this study, we utilize a deep convolutional neural network (Deep CNN) for the data-driven statics modeling and control of soft continuum robots. Once trained, the CNN model establishes a nonlinear function to handle input data, providing solutions across diverse inputs. The training and validation datasets are rooted in the Absolute Nodal Coordinate Formulation (ANCF) framework, as detailed in the research by Nada et al. [37], Shabana [38,39,40], and Taylor [41]. This formulation takes into account both geometric and material non-linearities within the multi-body dynamic system, thus presenting a closer-to-reality portrayal. Significantly, the beam element informed by ANCF moves beyond the assumption of a rigid cross-section, factoring in distortional cross-sectional deformations. This results in a holistic model for robotic structures, as showcased by Wang et al. [42].

The contribution of this research is leveraging the strength of CNN-based models in computational efficiency and precision, thus addressing a critical limitation of using ANCF alone. This integration allows for finding solutions to the inverse statics problem, which was previously challenging to resolve analytically by the ANCF. By integrating Deep CNN with ANCF, our approach represents a significant advancement over existing methods, providing a more robust and efficient tool for statics modeling in soft continuum robotics, while allowing for the solving of the inverse statics for soft robots modeled via ANCF. In this setup, the model receives a target end-effector position and endeavors to calculate the necessary external moments at the robot’s tip to reach the given position. Relying solely on ANCF modeling makes addressing the inverse statics issue quite complex. However, by incorporating data-driven, model-free control methods, there is an opportunity to tap into the complete capabilities of continuum robots across various applications. To assess and oversee the effectiveness of the updated CNN model, we employ a k-fold cross-validation method to gauge the performance of various models.

This paper is organized as follows: Section 2 delves into the derivation of the Absolute Nodal Coordinate Formulation (ANCF), showing its significance in multibody system modeling concerning continuum robots. Section 3 elaborates on the CNN quasi inverse static modeling of the continuum robot within the ANCF framework, discussing data gathering and model architectures. Subsequently, Section 4 showcases the results from our data-driven statics modeling and control techniques, evaluating their efficacy and considering potential future research directions. Finally, Section 5 recapitulates the main findings of the paper, emphasizing the importance of data-driven statics modeling and control in the context of soft continuum robots.

## 2. Absolute Nodal Coordinate Formulation (ANCF)

**Figure 1:**
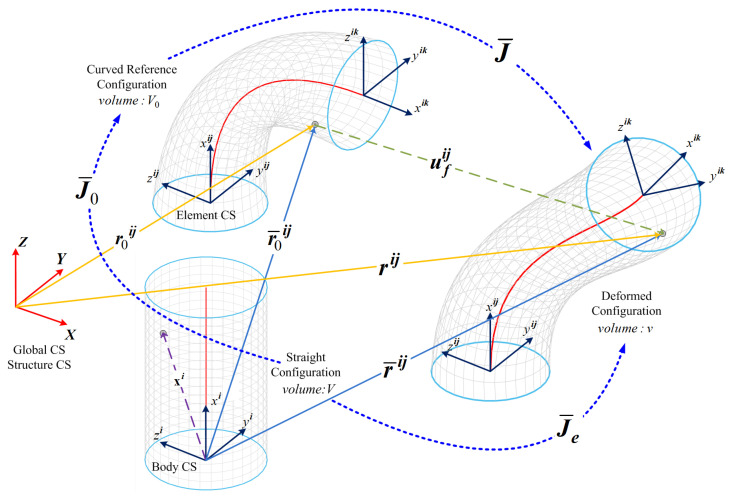
Diverse deformations exhibited by a 3D beam as represented by the ANCF.

In this section, we detail the steps of deriving the forward static modeling of the continuum robots based on the Absolute Nodal Coordinate Formulation (ANCF). The ANCF is a fundamental methodology in the realms of multi-body dynamics and finite element analysis, particularly adept at modeling structures undergoing significant deformations and complex geometries. This formulation is instrumental in characterizing the kinematics of intricate structures, such as flexible bodies, cables, and continuous systems, marking a departure from the conventional assumption of rigid cross-sections. This shift is crucial for accurately depicting the deformations experienced by continuum robots’ cross-sections during dynamic analyses. For instance, as depicted in Figure 1, a soft beam element with a circular cross-section connecting nodes 1 and 2 demonstrates complex deformations including spatial bending, twisting, and stretching. Leveraging this, the current research endeavors to develop an ANCF-based model for continuum robots, which is further refined through approximation using Deep Convolutional Neural Network (CNN) models. This approach ensures the incorporation of all relevant deformations, thus enhancing the fidelity and applicability of the model in dynamic scenarios.

### 2.1. Problem Definition

In the ANCF framework, absolute coordinates are employed to describe the deformation and motion of individual nodes within a structure, in contrast to local or relative coordinates. This use of absolute coordinates establishes a global reference frame, ensuring a more precise representation of node positions and orientations. This global reference system is referred to as the Structure Coordinate System {SCS: $\left(XYZ\right)$}, as introduced in [43].

For a two-noded beam element, as illustrated in Figure 1, the absolute nodal coordinates of a node *k*, where k=(1,2), situated in element *j* on body *i*, can be described by the following expression:

(1) $${\bar{e}}^{ijk}={\left[\begin{array}{cccc} {\bar{r}}^{ijk^{T}} & {\frac{\partial {\bar{r}}^{ijk}}{\partial x^{i}}}^{T} & {\frac{\partial {\bar{r}}^{ijk}}{\partial y^{i}}}^{T} & {\frac{\partial {\bar{r}}^{ijk}}{\partial z^{i}}}^{T} \end{array}\right]}_{12\times 1}^{T}$$

The spatial position of node *k* within the given framework is denoted by ${\bar{r}}^{ijk}$, while the gradients at node *k* with respect to the body coordinate system {BCS: $\left(x^{i}\;y^{i}\;z^{i}\right)$} are given by $\partial {\bar{r}}^{ijk}/\partial x^{i}$, $\partial {\bar{r}}^{ijk}/\partial y^{i}$, and $\partial {\bar{r}}^{ijk}/{\partial z}^{i}$. The BCS is aligned with the x-axis of the first element (j=1) of the beam mesh, represented by $\left(x^{i1}\right)$. Hence, no rotation transformation is needed between the BCS and the element coordinate system {ECS: ($x^{ij},y^{ij},z^{ij}$)} at the first element of the beam mesh. The vector $\partial {\bar{r}}^{ij}/\partial x^{i}$ defines the orientation of the beam’s centerline to the BCS, while the vectors $\partial {\bar{r}}^{ij}/{\partial y}^{i}$ and $\partial {\bar{r}}^{ij}/{\partial z}^{i}$ define the orientation of the height and width coordinates of the beam’s cross-section, respectively. These vectors may not be orthogonal unit vectors, as discussed in [40].

The transformation between the BCS: $\left(x^{i}y^{i}z^{i}\right)$ and the SCS:$\left(XYZ\right)$ can be expressed by a constant transformation matrix $A_{o}$, that is composed of the orthogonal unit vectors along the body reference frame, such that

(2) $$A_{o}=\frac{\partial X}{\partial x^{i}}$$

For the beam represented in Figure 1, assuming no translation is carried out, $A_{o}\in R^{3\times 3}$ can be expressed as a pure rotation matrix

(3) $$A_{o}=\left[\begin{array}{ccc} X\cdot x^{i} & X\cdot y^{i} & X\cdot z^{i}\\ Y\cdot x^{i} & Y\cdot y^{i} & Y\cdot z^{i}\\ Z\cdot x^{i} & Z\cdot y^{i} & Z\cdot z^{i} \end{array}\right]=\left[\begin{array}{ccc} 0 & 0 & -1\\ 0 & 1 & 0\\ 1 & 0 & 0 \end{array}\right]$$

Thus, the absolute nodal coordinates of the node *k* with respect to the **SCS** could be obtained with the transformation carried out, as $e^{ijk}=T_{o}{\bar{e}}^{ijk}$, such that

$$e^{ijk}=\left[\begin{array}{c} r^{ijk}\\ \frac{\partial r^{ijk}}{\partial X}\\ \frac{\partial r^{ijk}}{\partial Y}\\ \frac{\partial r^{ijk}}{\partial Z} \end{array}\right]=\left[\begin{array}{cccc} A_{o} & 0 & 0 & 0\\ 0 & A_{o} & 0 & 0\\ 0 & 0 & A_{o} & 0\\ 0 & 0 & 0 & A_{o} \end{array}\right]\left[\begin{array}{c} {\bar{r}}^{ijk}\\ \frac{\partial {\bar{r}}^{ijk}}{\partial x^{i}}\\ \frac{\partial {\bar{r}}^{ijk}}{\partial y^{i}}\\ \frac{\partial {\bar{r}}^{ijk}}{\partial z^{i}} \end{array}\right]$$

In this context, ${\bar{e}}^{ijk}$ and $e^{ijk}$ denote the nodal coordinates defined within the (BCS) and the (SCS), respectively. This choice of representation ensures the maintenance of inter-element continuity for global displacement gradients at these specific points. The nodal coordinates for an element consisting of two nodes can be succinctly expressed as a vector of

$${\bar{e}}^{ij}={\left[\begin{array}{cc} {\bar{e}}^{ij1T} & {\bar{e}}^{ij2T} \end{array}\right]}_{24\times 1}^{T}.$$

Therefore, in the ANCF, the position of an arbitrary point on the body *i*, element *j*, ${\bar{r}}^{ij}$ with respect to **BCS** $\left(x^{i}y^{i}z^{i}\right)$, and $r^{ij}$ with respect to **SCS** $\left(XYZ\right)$, can be defined as follows:

(4)r¯iju¯ij,t=Siju¯ije¯ijt(5)riju¯ij,t=Siju¯ijToTeijt(6)=Aor¯iju¯ij,t

such that $e^{ijk}=T_{o}{\bar{e}}^{ijk},$ and $S^{ij}$ is the element shape function matrix, and

$${\bar{u}}^{ij}={\left[\begin{array}{ccc} x^{ij} & y^{ij} & z^{ij} \end{array}\right]}^{T}$$

is the local position of the arbitrary point with respect to the **ECS,** where, $x^{ij},y^{ij}$, and $z^{ij}$ are the coordinates along the element *j*. A straightforward procedure to construct the shape function is demonstrated by [42]. Equation (5) is crucial when the nodal coordinates are defined using the structural coordinates system, while Equation (6) is used in the simulation of the output results on the global (structural) coordinate system.

The initial configuration is defined such that the time is equal to zero; thus, the absolute position vector of an arbitrary point in the reference configuration can be described as

(7) $${\bar{r}}^{ij}\left({\bar{u}}^{ij},0\right)={\bar{r}}_{0}^{ij}=S^{ij}\left({\bar{u}}^{ij}\right){\bar{e}}_{0}^{ij}$$

where ${\bar{e}}_{0}^{ij}$ is the vector of nodal coordinates in the reference configuration with respect to the body coordinate system (**BCS**).

The displacement field, ${\bar{r}}^{ij}\left({\bar{u}}^{ij},t\right)$, can be written as

(8) $${\bar{r}}^{ij}\left({\bar{u}}^{ij},t\right)={\bar{r}}_{0}^{ij}+u_{f}^{ij}=S^{ij}\left({\bar{u}}^{ij}\right)\left({\bar{e}}_{0}^{ij}+{\bar{e}}_{f}^{ij}\right)$$

where ${\bar{e}}_{f}^{ij}$ is the vector of nodal displacements with respect to the **BCS**. It can be shown that a line element $dx^{i}$ in the straight configuration corresponds to a line element $d{\bar{r}}_{0}^{ij}$ in the initial configuration and to a line element $d{\bar{r}}^{ij}$ in the current configuration. One has the following relationships:

$$d{\bar{r}}^{ij}={\bar{J}}^{ij}d{\bar{r}}_{0}^{ij}\;\;\;,d{\bar{r}}^{ij}={\bar{J}}_{e}^{ij}dx^{i}\;\;\;,d{\bar{r}}_{0}^{ij}={\bar{J}}_{0}^{ij}dx^{i}$$

The gradients of the displacement vector, matrix $J^{ij}$, are defined as follows:

(9) $${\bar{J}}^{ij}=\frac{\partial {\bar{r}}^{ij}}{\partial {\bar{r}}_{0}^{ij}}=\frac{\partial {\bar{r}}^{ij}}{\partial x^{i}}\frac{\partial x^{i}}{\partial {\bar{r}}_{0}^{ij}}={\bar{J}}_{e}^{ij}{\bar{J}}_{0}^{ij-1}$$

If the element local coordinate system is parallel to the body coordinate system and the element is not curved, matrix $J_{0}^{ij}$ is an identity matrix. The relationship between the volume of the curved structure $V_{o}$ in the initial configuration, to the volume of the straight configuration *V* is defined as

$$\begin{array}{ccc} dV_{o} & = & \left|{\bar{J}}_{0}\right|dV\\ dv & = & \left|{\bar{J}}_{e}\right|dV \end{array}$$

Note that

$${\bar{J}}_{0}=\frac{\partial {\bar{r}}_{0}^{ij}}{\partial x^{i}}=\frac{\partial S^{ij}\left({\bar{u}}^{ij}\right)}{\partial x^{i}}{\bar{e}}_{0}^{ij}=\frac{\partial S^{ij}\left({\bar{u}}^{ij}\right)}{\partial x^{i}}T_{o}^{T}e_{0}^{ij}$$

where $\left|{\bar{J}}_{0}\right|$ is the determinant of the matrix of position vector gradients ${\bar{J}}_{0}$ , which is constant. By defining $p^{i}$ as the unconstrained (free) vector of nodal coordinates over the flexible body *i* with the dimension of DOFs×1, DOFs are the total number of degrees of freedom. Thus, Equation (4) can be rewritten as follows:

(10) $${\bar{r}}^{ij}=S^{ij}{\bar{e}}^{ij}=S^{ij}B_{1}^{ij}B_{2}^{i}{\bar{p}}^{i}$$

where $B_{1}^{ij}$ is the connectivity matrix and $B_{2}^{i}$ is boundary conditions linear-transformation matrix.

### 2.2. Elastic Forces

The nonlinear Lagrangian strain tensor, $\epsilon_{L}$, can be defined using the right Cauchy–Green deformation tensor as follows [39]:

(11) $$\epsilon_{L}=\frac{1}{2}\left(J^{T}J-I\right)$$

The gradients of the displacement vector are defined in Equation (9), in which $J=\partial r^{ij}/\partial r_{0}^{ij}=J_{e}J_{0}^{-1}$. It is possible to verify that, if the body experiences a rigid motion, matrix J is orthonormal, and thus it is clear from Equation (10) that there is no strain. Note that

$$J_{e}=\frac{\partial r^{ij}}{\partial X}=\frac{\partial A_{o}{\bar{r}}^{ij}}{\partial x^{i}}\frac{\partial x^{i}}{\partial X}=A_{o}\frac{\partial {\bar{r}}^{ij}}{\partial x^{i}}A_{o}^{T}=A_{o}{\bar{J}}_{e}A_{o}^{T}$$

Similarly, $J_{0}=A_{o}{\bar{J}}_{0}A_{o}^{T}$, thus

(12) $$J=A_{o}{\bar{J}}_{e}{\bar{J}}_{0}^{-1}A_{o}^{T}=A_{o}{\bar{J}}^{ij}A_{o}^{T}$$

where ${\bar{J}}^{ij}=\partial {\bar{r}}^{ij}/\partial {\bar{r}}_{0}^{ij}$, is defined in Equation (9). Therefore, the strain tensor with respect to the element coordinate system can be defined as

(13) $$\begin{array}{ccc} \epsilon_{L} & = & A_{o}{\bar{\epsilon }}_{L}A_{o}^{T} \end{array}$$

(14) $$\begin{array}{ccc} {\bar{\epsilon }}_{L} & = & \frac{1}{2}\left({\bar{J}}^{T}\bar{J}-I\right) \end{array}$$

Because of the symmetry of the strain tensor, it is sufficient to identify only six strain components, three normal strains, and three shear strains, such that the strain vector can be written as

(15) $$\epsilon ={\left[\begin{array}{cccccc} \epsilon {}_{xx} & \epsilon {}_{yy} & \epsilon {}_{zz} & 2\epsilon {}_{xy} & 2\epsilon {}_{xz} & 2\epsilon {}_{yz} \end{array}\right]}^{T}$$

The stress components of the element can be defined using the constitutive equation as follows

(16) σ=Eϵ

where E is the matrix of the elastic constants of the material [44]. The elastic forces of the element can be derived by using the following expression of the strain energy:

(17) $$U=\frac{1}{2}\int_{V}\epsilon^{T}\sigma \;dV=\frac{1}{2}\int_{V}\epsilon^{T}E\epsilon \;dV$$

The vector of the elastic forces, $Q_{k}^{ij}$ , that is produced due to deformation of element *j*, can be defined using the strain energy, *U*, as follows:

(18) $$Q_{k}^{ij}=\frac{\partial U^{ij}}{e^{ij}}$$

Several studies investigated the accuracy and usability of a continuum mechanics approach in the description of elastic forces of a three-dimensional beam element [45,46,47]. It should be mentioned that the strain tensor given by Equation (14) is defined as a function of the element coordinates ECS$\left(x^{ij}y^{ij}z^{ij}\right)$ and, therefore, the elastic forces are defined as a volume element that allows for describing the deformation of the beam cross-section.

### 2.3. Generalized External Forces

The principle of virtual work can be used to develop the vector of the generalized forces on the body *i*, element *j*, i.e., $Q_{e}^{ij}$, that can be developed due to external Cartesian forces and moments. The virtual work carried out by applying an external force vector, acting on an arbitrary point on the element, can be written with respect to SCS $\left({\;F}^{ij}\right)$ or BCS $\left({\bar{F}}^{ij}\right)$ as follows

(19) $$\delta W={\bar{F}}^{ijT}\delta {\bar{r}}^{ij}=Q_{e}^{ijT}\delta e^{ij}$$

Substituting the value of $\bar{r}$ from Equation (5) yields

$$\begin{array}{ccc} \delta W & = & {\bar{F}}^{ijT}\delta {\bar{r}}^{ij}\\ & = & {\bar{F}}^{ijT}S^{ij}\delta {\bar{e}}^{ij}\\ & = & {\bar{F}}^{ijT}S^{ij}T_{o}^{iT}\delta e^{ij} \end{array}$$

(20) $$Q_{e}^{ijT}={\bar{F}}^{ijT}S^{ij}T_{o}^{iT}$$

In the case of describing the generalized forces related to the unconstrained nodal coordinates of the body $\left(i\right)$, i.e., $p^{i}$, one can conclude that

(21)δW=F¯ijTSijToiTB1ijB2iδpi⇕(22)QpiT=F¯ijTSijToiTB1ijB2i

The absence of rotating angular coordinates in ANCF elements complicates the application of moments compared to the application of point forces. In the context of applied moments, it can be held that

(23) $$\delta W={\bar{M}}^{ijT}\delta {\bar{\vartheta }}^{ij}$$

which is the applied moment ${\bar{M}}^{ij}$ at a given point on the body (i)-element (j) times the virtual change in orientation $\delta {\bar{\vartheta }}^{ij}$, at that point. The virtual change $\delta {\bar{\vartheta }}^{ij}$ can be expressed as

(24) $$\delta {\bar{\vartheta }}^{ij}={\bar{\omega }}^{ij}\delta t$$

Therefore, the formulation of the virtual change in orientation $\delta {\bar{\vartheta }}^{ij}$ can be obtained when the angular velocity for a material point, ${\bar{\omega }}^{ij}$, inside an ANCF element is defined. The vorticity tensor describes the angular velocity ${\bar{\omega }}^{ij}$ at the given point in the continuum element and can be expressed as follows [48]:

(25) $${\tilde{\bar{\omega }}}^{ij}=\left[\begin{array}{ccc} 0 & -{\bar{\omega }}_{z} & {\bar{\omega }}_{y}\\ {\bar{\omega }}_{z} & 0 & -{\bar{\omega }}_{x}\\ -{\bar{\omega }}_{y} & {\bar{\omega }}_{x} & 0 \end{array}\right]=\frac{1}{2}\left(L-L^{T}\right)$$

where L is the velocity gradient tensor that can be defined as

(26) $$L=\frac{\partial {\dot{\bar{r}}}^{ij}}{\partial {\bar{r}}^{ij}}$$

Equation (26) can be written in terms of the deformation gradient as follows:

(27) $$L=\frac{\partial {\dot{\bar{r}}}^{ij}}{\partial {\bar{r}}^{ij}}=\frac{\partial {\dot{\bar{r}}}^{ij}}{\partial {\bar{r}}_{0}^{ij}}\frac{\partial {\bar{r}}_{0}^{ij}}{\partial {\bar{r}}^{ij}}=\;{\dot{\bar{J}}}^{ij}{\left({\bar{J}}^{ij}\right)}^{-1}$$

Substituting the value of ${\bar{J}}^{ij}$, Equation (9), yields

(28) $$L={\dot{\bar{J}}}_{e}^{ij}{\bar{J}}_{0}^{ij^{-1}}\;{\bar{J}}_{0}^{ij}\;{\bar{J}}_{e}^{ij^{-1}}={\dot{\bar{J}}}_{e}^{ij}{\bar{J}}_{e}^{ij^{-1}}$$

It can be seen that the velocity gradient L can now be written in terms of the nodal coordinates and the time derivative of the nodal coordinates. Using Equation (25), one can conclude that

(29) $${\bar{\omega }}^{ij}=\left[\begin{array}{c} {\bar{\omega }}_{x}\\ {\bar{\omega }}_{y}\\ {\bar{\omega }}_{z} \end{array}\right]=\frac{1}{2}\left[\begin{array}{c} L_{3,2}-L_{2,3}\\ L_{1,3}-L_{3,1}\\ L_{2,1}-L_{1,2} \end{array}\right]={\bar{G}}^{ij}\dot{\bar{e}}$$

Substituting the value of ${\bar{\omega }}^{ij}$ into Equation (24), and then into the virtual work of Equation (23), yields

(30)δW=M¯ijTω¯ijδt(31)=M¯ijTG¯ije¯˙ijδt(32)=M¯ijTG¯ijδe¯ij(33)=M¯ijTG¯ijToiTδeij

Thus, the generalized forces due to external moments can be described as follows:

(34) $$\begin{array}{ccc} Q_{e}^{ijT} & = & {\bar{M}}^{ijT}\;{\bar{G}}^{ij}\;T_{o}^{iT} \end{array}$$

(35) $$\begin{array}{ccc} Q_{p}^{ijT} & = & {\bar{M}}^{ijT}\;{\bar{G}}^{ij}\;T_{o}^{iT}B_{1}^{ij}B_{2}^{i} \end{array}$$

The complete vector of the generalized forces, due to external forces and moments can be written as follows:

(36) $$Q_{p}^{ijT}=\left({\bar{F}}^{ijT}S^{ij}+{\bar{M}}^{ijT}\;{\bar{G}}^{ij}\right)\;T_{o}^{iT}B_{1}^{ij}B_{2}^{i}$$

### 2.4. Static Analysis

The equations of motion of the continuum robot structure, say, body i, can then be written in a matrix form in terms of the unconstrained generalized coordinates $\ddot{p}$ as

(37) $$M^{i}{\ddot{p}}^{i}=Q_{k}^{iT}+Q_{p}^{iT}=Q^{iT}$$

Note the complete set of generalized coordinates of the body, $e^{i}=B_{1}^{ij}B_{2}^{i}p^{i}$. The $Q_{p}^{i}$ is the vector of generalized external forces and moments that was discussed in the last section (Equation (36)) and $Q_{k}^{i}$ is the elastic forces, as expressed in Equation (18). The preceding equation accounts for all geometric nonlinearities since nonlinear strain-displacement relations are used (Equation (14)). Thus, the static analysis can be carried out by solving the following equation:

(38) $$Q^{i}\left(p\right)=0$$

**Figure 2:**
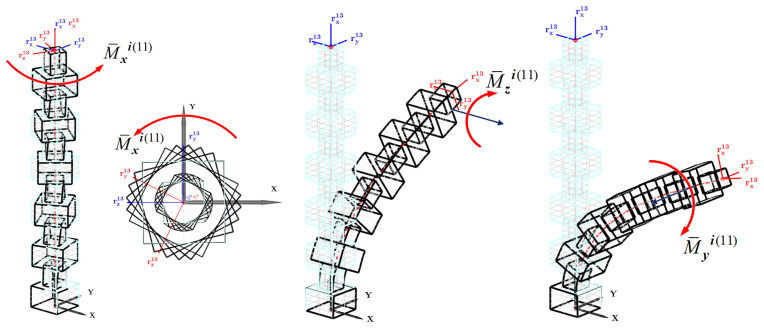
Effect of applying moments on continuum robot structure.

The nonlinear equations can be solved using the Newton–Raphson iteration procedure in which the values of the nodal coordinates at step $\left(n+1\right)$ can be calculated as

(39) $$\begin{array}{ccc} p^{\left(n+1\right)} & = & p^{\left(n\right)}+\Delta p \end{array}$$

(40) $$\begin{array}{ccc} \Delta p & =- & {\left[\frac{\partial Q^{i^{\left(n\right)}}}{\partial p^{\left(n\right)}}\right]}^{-1}Q^{i^{\left(n\right)}} \end{array}$$

where $\partial Q^{i^{\left(n\right)}}/\partial p^{\left(n\right)}$ is called a tangent stiffness matrix and vector $Q^{i^{\left(n\right)}}$ includes the elastic and external forces at iteration step *n*. In the solution process, the tangent stiffness matrix is calculated numerically using perturbations in the nodal coordinates and finite differences. The convergence criterion for the iteration is defined as follows:

(41) $$\left|\Delta p\right|<\varepsilon$$

where $\left|.\right|$ is the Euclidean norm of the vector. Thus, given the Cartesian forces and/or moments, the generalized forces can be calculated, and the corresponding nodal coordinates can be obtained. Figure 2 shows the various configurations of continuum robots, whereby Cartesian forces are applied along the local *x*- and *z*- axes, and Cartesian moments are applied about the *z*- and *y*- axes to excite bending modes, where 12 elements of silicone rubber are forming the continuum robot. The last-node frame representing the tip point orientation (node 13) of both the un-deformed and deformed shapes are shown. The figures demonstrate the robot connectivity and continuity, while also validating the correctness of the elastic and external force formulation of the beam element. It is crucial to note that the derivation of the velocity transformation matrix ${\bar{G}}^{ij}\left(e\right)$, which depends on the nodal coordinates, is significant in the context of applying moments to a cross-section undergoing rotation. The subsequent derivation of the elements of this matrix will be elaborated upon in the inverse solution.

### 2.5. 3D-Beam Element with Circular Cross Section

**Figure 3:**
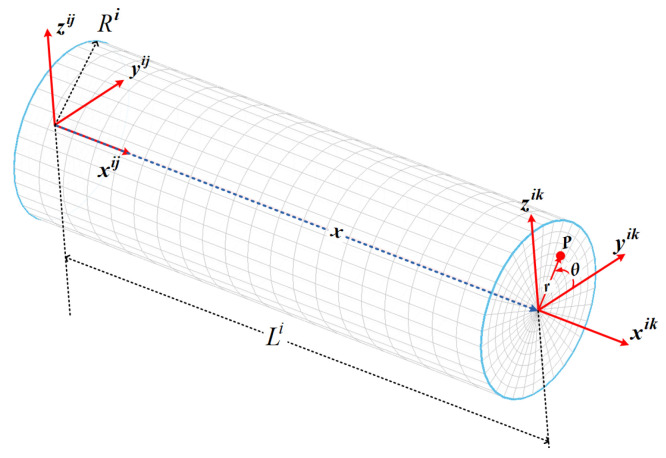
Beam element of circular cross section.

A well-known structure of a 3D beam with a circular cross section, i.e., circular rods, is utilized in constructing continuum robots. In this case, the coordinates of the material point in the undeformed configuration with respect to the element frame are described in terms of the axial and radial displacements and an angle measured counter clockwise from the element frame. The coordinates of an arbitrary point can be described as ${\bar{\varrho }}^{ij}:{\left[\begin{array}{ccc} x^{ij} & r^{ij} & \theta^{ij} \end{array}\right]}^{T}$. The Cartesian coordinates are related to the cylindrical coordinates (see Figure 3), by

(42) $$\left.\begin{array}{c} x=x\\ y=r\cos\left(\theta \right)\\ z=r\sin\left(\theta \right) \end{array}\right\}$$

Using Equation (42), the shape function can be described by the cylindrical coordinates, i.e., $S^{ij}\left({\bar{u}}^{ij}\right)\to S^{ij}\left({\bar{\varrho }}^{ij}\right)$. The transformation of the displacement field can be carried out as

(43) $$\left[\begin{array}{c} {\bar{u}}_{x}\\ {\bar{u}}_{y}\\ {\bar{u}}_{z} \end{array}\right]=\left[\begin{array}{ccc} 1 & 0 & 0\\ 0 & \cos\theta & -\sin\theta \\ 0 & \sin\theta & \cos\theta \end{array}\right]\left[\begin{array}{c} {\bar{u}}_{x}\\ {\bar{u}}_{r}\\ {\bar{u}}_{\theta } \end{array}\right]$$

The Jacobian matrix can be described in cylindrical coordinates $\varrho :\left(x,r,\theta \right)$ as follows:

(44) $$\bar{D}=\frac{\partial {\bar{u}}^{ij}}{{\bar{\varrho }}^{i}}=\left[\begin{array}{ccc} \frac{\partial {\bar{u}}_{x}}{\partial x} & \frac{\partial {\bar{u}}_{x}}{\partial r} & \frac{\partial {\bar{u}}_{x}}{\partial \theta }\\ \frac{\partial {\bar{u}}_{r}}{\partial x} & \frac{\partial {\bar{u}}_{r}}{\partial r} & \frac{\partial {\bar{u}}_{r}}{\partial \theta }\\ \frac{\partial {\bar{u}}_{\theta }}{\partial x} & \frac{\partial v}{\partial r} & \frac{\partial {\bar{u}}_{\theta }}{\partial \theta } \end{array}\right]$$

Thus, the gradients of the displacement vector can be estimated as follows [49]:

(45) $${\bar{J}}_{e}^{ij}=\left[\begin{array}{ccc} {\bar{D}}_{1,1} & {\bar{D}}_{2,1} & \frac{1}{r}{\bar{D}}_{3,1}\\ {\bar{D}}_{2,1} & {\bar{D}}_{2,2} & \frac{1}{r}\left({\bar{D}}_{2,3}-{\bar{u}}_{\theta }\right)\\ {\bar{D}}_{3,1} & {\bar{D}}_{3,2} & \frac{1}{r}\left({\bar{D}}_{3,2}+{\bar{u}}_{r}\right) \end{array}\right]$$

A similar procedure can be carried out to estimate ${\bar{J}}_{0}^{ij}$, and then the deformation gradients ${\bar{J}}^{ij}$ (Equation (9)) can be calculated. Based on the deformation gradient, the strain tensor of a beam element with a circular cross area can be calculated using Equation (14). The axial component $\epsilon_{xx}$ remains the same as for a rectangular cross section. Moreover, the variables of volume integration are the parameters $\left(x,r,\theta \right)$ and the volume integration boundaries are altered to $V^{ij}=$$\int_{0}^{L}\cdots \int_{-\pi }^{+\pi }\cdots \int_{0}^{R}\cdots rdrd\theta dx$. Using the mapped forms described above, the dynamic terms can be updated for circular cross section 3D beams.

## 3. Deep-CNN Quasi-Statics Modeling

The primary objective of developing a deep CNN model is to construct a surrogate model that can effectively approximate the challenging inverse problem of ANCF statics modeling of continuum robots. The CNN model is intended to determine immediate solutions for the tip moments ${\bar{M}}^{i\;13}$ that are necessary to achieve a specific tip pose, as defined by $p\in R^{12}$. In the current study, our focus was on the effects of constant moments applied to the robot tip. These moments, which could be implemented via applying tensions to cables in a cable-driven continuum robot, primarily induce pure bending. This type of bending is less likely to result in buckling deformation within the cross-section of the robot.

The primary distinction of the CNN utilized in our research lies in its inherent ability to effectively capture and analyze spatial correlations within the dataset. This aspect is particularly advantageous in our application, where the key features include the position and orientation of the robot’s tip. These features are inherently spatially correlated across the robot’s workspace, largely due to the physical constraints and specific dimensions of the robot. By doing so, the model can efficiently predict the required tip moments, thereby solving the inverse problem.

### 3.1. Data Collection and Processing

Data-driven modeling can be enhanced by increasing the size of the training dataset, resulting in more accurate predictions. However, this comes at the expense of substantial computational resources. Our research integrated the ANCF model, obtained in Equation (38), to generate the training datasets. We applied a set of moments $M=[M_{x},M_{y},M_{z}]$ to the robot’s end-effector into the ANCF model and derived the robot’s corresponding poses $p\in R^{12}$ containing the position and gradients. The three moment values are swept one after the other across the entire range of the robot’s workspace. This dataset forms the foundation for our data-driven approach to model the robot’s behavior.

**Figure 4:**
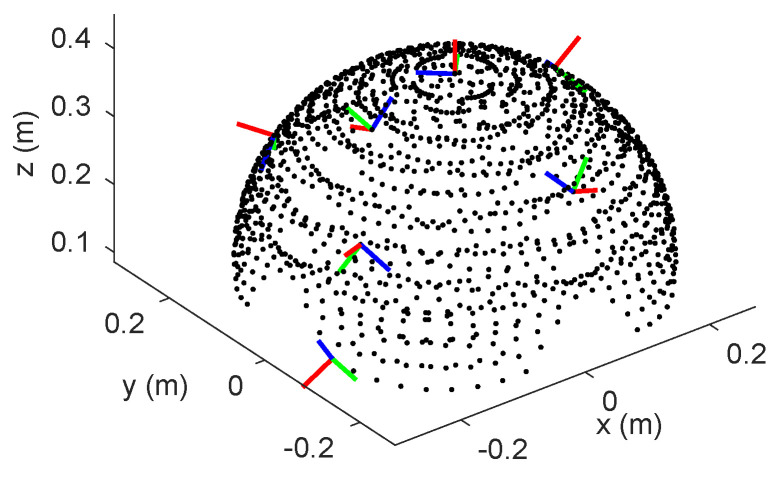
Sampled data points from the robot’s workspace were used as the training dataset for the CNN-based static modeling. The orientation of the robot’s end-effector is displayed as coordinate frames at selected data points, where the red, green, and blue axes represent the *x*, *y*, and *z* coordinates respectively.

It can be quite time-consuming to explore all the possible combinations of moments for the robot’s tip. As a pragmatic approach, we generated a comprehensive dataset with 1520 data points. The collected data points were obtained within the expected workspace of the continuum robot, which has a fixed length of 40 cm. The sampling process was meticulously designed to ensure thorough coverage of the operational range of the robot, while also adhering to permissible moment limits, as depicted in Figure 4, showing the positions and orientations of the robot’s end-effector. In order to ensure reliable model performance and generalization, the dataset was divided into three subsets: 70% for training, 15% for validation, and the remaining 15% for rigorous testing. This partitioning strategy was implemented to aid in achieving robust model training and evaluation.

**Figure 5:**
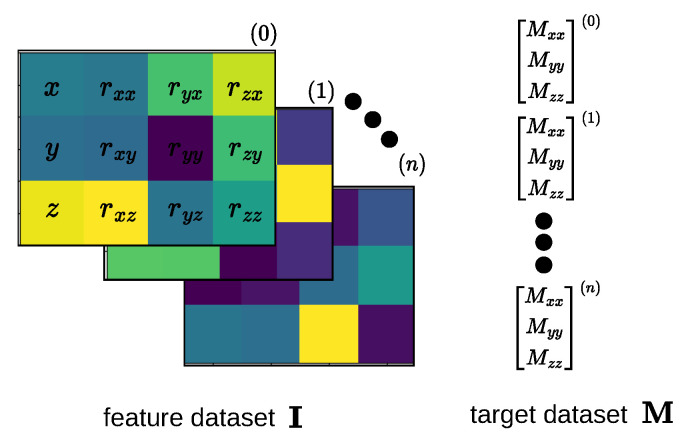
Stacking of robot’s position and gradients to form a 3×4 image representing the robot’s pose to be used in the CNN-based statics modeling of a continuum robot.

In order to utilize CNNs for modeling the statics of the continuum robot, we must first convert each data point, represented as $p_{i}\in R^{12}$, into an image. This is accomplished by stacking the robot tip position in the *x*, *y*, and *z* dimensions with the gradient vectors *r* as detailed in Figure 5. The resulting feature images, which represent the robot’s pose, are denoted as $I\in R^{1520\times 3\times 4}$. To enhance the model’s training stability, the training and testing data are normalized using the Z-score technique, which ensures that our data have a mean of μ=0 and a standard deviation of σ=1. Each data point p is normalized as follows:

p′=(p−μ)/σ

It is important to note that the values of μ and σ are calculated solely based on the training data.

### 3.2. CNN Model Architectures

**Figure 6:**
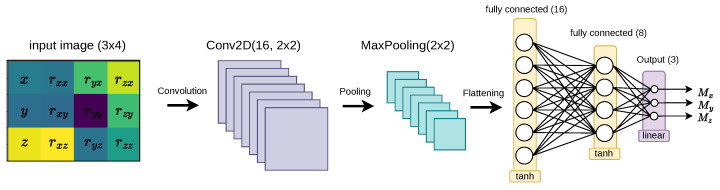
The CNN architectures employed in the inverse statics modeling of continuum robots.

In this document, we present a CNN framework that has been specifically designed to approximate the inverse statics of the robot modeled using the ANCF. The input size of the network is fixed at 3×4, corresponding to the representative image of the tip’s pose, while the objective of the network is to predict the required tip moments as its output. The CNN model begins with a convolutional layer, pooling layer, and flatten layer, followed by feeding the input data to the fully connected layers, as shown in Figure 6. The kernel size of the first convolution layer is chosen to be 16 while the hyperbolic tangent (tanh) activation function is selected.

### 3.3. Training of DNN Models

In the course of training our CNN models, we employed the Mean-Squared Error (MSE) loss function. This choice is of utmost significance in training the deep regressor model that approximates the inverse statics of the continuum robot. The MSE loss function effectively quantifies the divergence between the CNN model’s estimated output, denoted as $\hat{y}\left(\zeta \right)$, and the corresponding reference output, *y*, which relates to either forward or inverse statics modeling. The Mean-Squared Error is expressed mathematically as follows:

(46) $$\mathrm{MSE}=\frac{1}{N}\sum_{i=0}^{N-1}{\left[\hat{y}{\left(\zeta \right)}^{i}-y^{i}\right]}^{2}$$

The ζ symbol in the given equation denotes the network parameters, encompassing learnable weights and biases. To enhance the model’s performance, the Adam optimizer [50] has been employed, with an initial learning rate of $10^{-2}$ as its hyperparameter. Furthermore, the customization of hyperparameters is warranted for individual numerical examples, such as the training batch size and the number of epochs, which will be explained in detail in the subsequent sections.

## 4. Results and Discussion

In this section, we conducted a series of numerical experiments to evaluate the effectiveness of the proposed CNN quasi-static models based on ANCF. Firstly, we assessed the performance of the CNN model by examining its training and validation losses throughout training, which allowed us to identify potential areas for improvement. Subsequently, we compared the trained model with the testing data collected, enabling us to establish its reliability and accuracy. Furthermore, we employed the proposed CNN-based inverse model to generate the moments required to track a specified trajectory in the task space, which yielded promising results. To determine the optimal model architecture, we conducted a five-fold cross-validation analysis on five models, enabling us to make informed decisions.

### 4.1. CNN Model Performance

**Figure 7:**
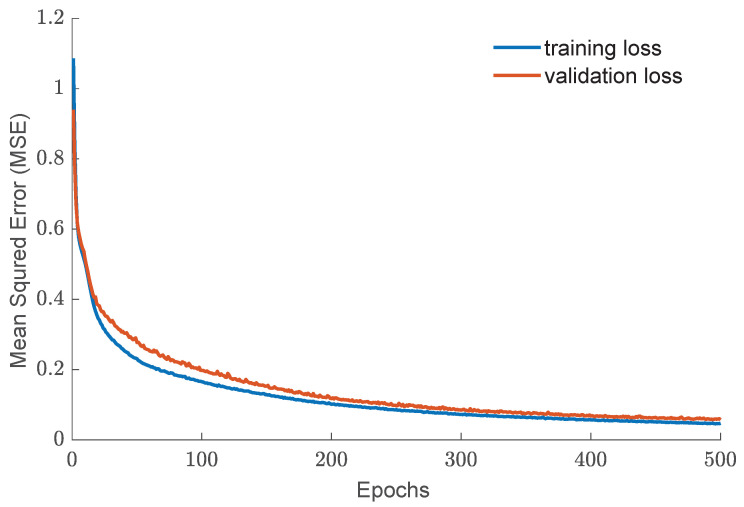
Graph showing the Mean Squared Error (MSE) of training and validation losses during the Convolutional Neural Network (CNN) training for the inverse static modeling of the continuum robot.

The TensorFlow 2.x API was used to execute the training and prediction processes on a personal computer. It is worth noting that the choice of computer hardware significantly impacts the duration of the training process. This study used a central processing unit (CPU) with a clock speed of 2.8 GHz to train the models. Figure 7 depicts the Mean Squared Error (MSE) losses for the CNN inverse statics models across 500 epochs and their convergence towards minimum loss values in the training and validation datasets.

**Figure 8:**
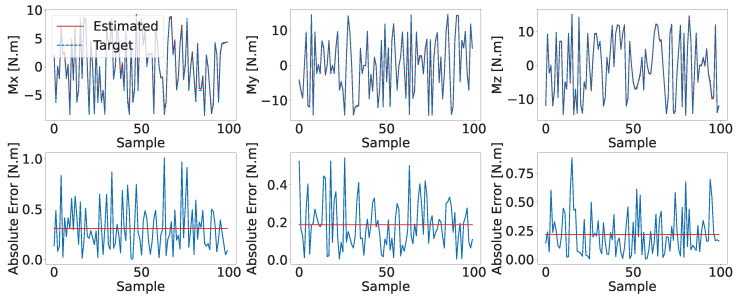
A comparison of the target versus estimated moments in the CNN-based inverse statics model, including the corresponding absolute error and the mean absolute errors highlighted with red horizontal lines.

To evaluate the performance of the models, the testing dataset consisting of 512 samples with a batch size of 64 is used. A comparison between the CNN-based model’s estimated and target data is represented in Figure 8, along with the corresponding absolute error. Due to space constraints, only the first 100 samples are displayed. Both models show a negligible error level, as evidenced by the close proximity between the estimated and target signals.

### 4.2. Trajectory Generation

**Figure 9:**
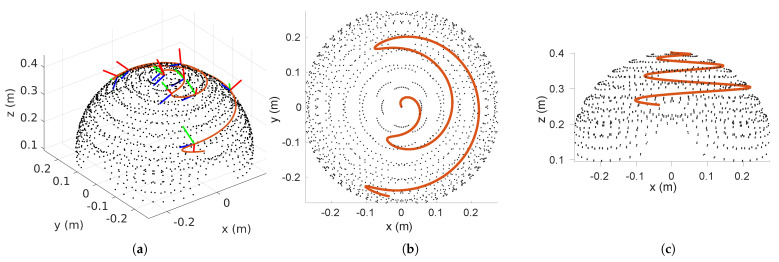
Reference trajectory generated to test the applicability of the proposed CNN inverse statics model of the continuum robot. (**a**) is showing the reference trajectory in orange over the dataset and the reference poses in RGB coordinates, (**b**,**c**) are the projection of the generated reference.

In order to evaluate the practicality of the proposed CNN-based quasi-inverse statics model, a reference trajectory has been provided, which specifies the desired poses over time for the robot’s tip to follow. The CNN model is responsible for computing the necessary moments to be applied at the robot’s tip to ensure it adheres to the reference trajectory. The reference trajectory is illustrated in Figure 9, highlighted in red and denoted as $p_{ref}\in R^{12}$ with the corresponding coordinates indicating the tip orientation. The reference trajectory is computed based on the Constant Curvature Model (CCM) [17], which is widely adopted in defining the kinematics of a single-segment continuum robot. The reference rotation trajectory $R_{\mathrm{ref}}$ and the reference positions $x_{\mathrm{ref}}$ are chosen as

(47) $$R_{\mathrm{ref}}=\left[\begin{array}{ccc} C_{\phi }^{2}\left(C_{\theta }-1\right)+1 & S_{\phi }C_{\phi }\left(C_{\theta }-1\right) & -C_{\phi }S_{\theta }\\ S_{\phi }C_{\phi }\left(C_{\theta }-1\right) & C_{\phi }^{2}\left(C_{\theta }-1\right)+1 & -S_{\phi }S_{\theta }\\ C_{\phi }S_{\theta } & S_{\phi }S_{\theta } & C_{\theta } \end{array}\right]$$

(48) $$x_{\mathrm{ref}}\left[\begin{array}{c} \frac{s\;C_{\phi }\left(C_{\theta }-1\right)}{\theta }\\ \frac{s\;S_{\phi }\left(C_{\theta }-1\right)}{\theta }\\ \frac{s\;S_{\theta }}{\theta } \end{array}\right]$$

where $S_{\left\{.\right\}}$ and $C_{\left\{.\right\}}$ represent the sine and cosine functions of an angle, respectively. The *s* symbol represents the robot length of 40 cm, while θ and ϕ are the curvature and plan of curvature angle that are swept to generate the reference trajectory within the robot’s workspace.

**Figure 10:**
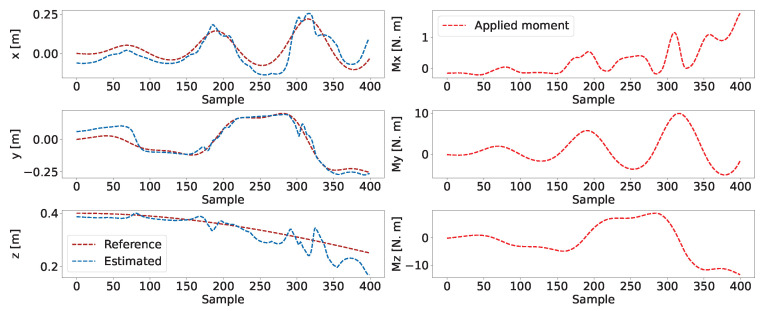
Comparative analysis of reference trajectories for CNN-based inverse statics testing versus trajectories derived from moments estimated by the CNN model.

The CNN-based inverse statics model processes the input reference trajectory to provide the corresponding moments to be applied. These moments are seamlessly integrated into the analytical model derived from ANCF, as expressed in Equation (39). Figure 10 compares the trajectories generated using the CNN-based approach and the model-based approach, along with the associated tip-moment profiles. The observed similarities between both trajectories indicate the immense potential of the developed CNN-based inverse model for trajectory generation and control. While the error may seem significant, we believe it to be acceptable for two key reasons. Firstly, the larger discrepancies are predominantly observed along the z-axis, specifically below 0.2 m. As illustrated in Figure 9, this region of the workspace contains relatively few data points. This scarcity is a consequence of the range of moments applied during the dataset generation, which inherently limits the precision of solutions in this area. Secondly, the reference trajectory was constructed using the constant curvature model, primarily to ensure realistic orientations for the desired trajectory. However, it is important to note that the constant curvature model does not necessarily align precisely with the general ANCF model, which may contribute to the observed deviations.

### 4.3. K-Fold Cross Validation

K-fold cross-validation is a robust method for assessing the performance and generalizability of predictive models, particularly in fields such as machine learning and statistical analysis [51]. In this technique, the dataset is divided into *k* equal or nearly equal-sized subsets or folds. The process involves using one of these folds as the validation set to test the model, and the remaining k−1 folds serve as the training set. This procedure is repeated *k* times, with each of the *k* subsets used exactly once as the validation data. The results from these *k* iterations are then averaged to produce a single estimation. This method is highly regarded for its ability to mitigate the bias associated with the random sampling of the training and validation sets, as every data point is in a validation set exactly once and in a training set k−1 times. It is particularly beneficial in situations where the available data are limited, as it maximizes both the training and testing data available for each iteration. By doing so, k-fold cross-validation provides a more comprehensive insight into the model’s performance, helping to identify issues such as overfitting or underfitting, and thus leading to more robust and reliable models.

In this subsection, the performance of the proposed CNN model was rigorously evaluated utilizing the K-Fold Cross-Validation method. This methodological approach is pivotal for extensively testing an inverse statics model across a range of architectural configurations, thereby ensuring their robustness and reliability. The dataset was partitioned into k=5 distinct subsets, facilitating an iterative process of training and evaluating the model. Such a comprehensive assessment strategy enables a deeper understanding of the model’s performance under diverse conditions. More critically, this evaluation framework not only aids in selecting the most efficacious architectural configuration but also offers insightful perspectives on the generalization capabilities of the model in realistic scenarios.

In this research, a series of five Deep Neural Network (DNN) models were developed, utilizing both feed-forward and Convolutional Neural Network (CNN) architectures. The models encompass a range of trainable parameters, varying from 259 to 2515, and are structured across two to three layers, as elaborated in Table 1. Convolutional layers within these models are indicated by the prefix ’C’, followed by a subscript denoting the number of kernels and the size. Fully connected layers are represented with the prefix ’F’, accompanied by a subscript indicating the number of neurons. For convolutional layers, a stride of 1 was consistently employed, without the inclusion of padding. The activation functions were predominantly set to the hyperbolic tangent (tanh), except for the output layer where a linear activation function was implemented. In scenarios where the model lacked a convolutional layer at the input stage, a flattened layer was integrated to transform the input pose image into a column vector.

**Figure 11:**
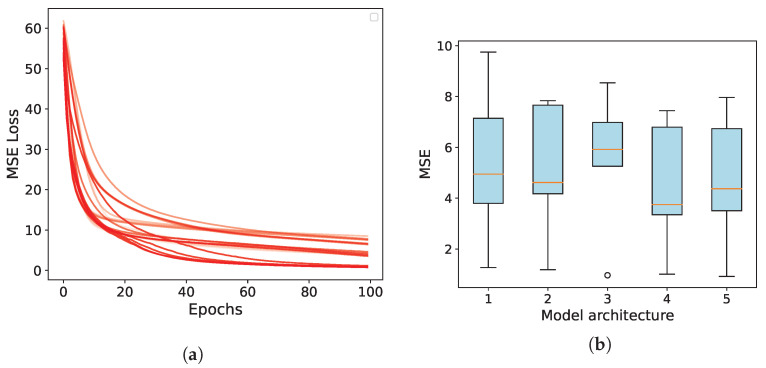
(**a**) Training losses expressed as mean squared error (MSE) for five deep neural network (DNN) models during five-fold cross-validation. (**b**) The mean and standard deviation of the MSE achieved by each model across the five folds.

The training of these models was executed using the Adam optimizer across 100 epochs, with Mean Squared Error (MSE) serving as the loss function. This process was further augmented by a five-fold cross-validation approach to ensure a comprehensive evaluation and validation. The convergence patterns of the learning algorithm, encompassing all networks and folds, are depicted in Figure 11a. Additionally, Figure 11b provides a detailed analysis of the mean values and standard deviations of the MSE losses for each model architecture, specifically focusing on the CNN inverse statics models.

Notably, networks with a greater number of parameters are represented in dark red, indicating that larger networks exhibit improved convergence towards smaller MSE values. To simplify our selection process, we opted for a neural network that performed reasonably well in both the training and testing phases. This exploration of compact and fast-learning architectures holds the promise of bolstering the overall system’s robustness.

## 5. Conclusions

In conclusion, this research introduces a data-driven modeling approach, employing a deep convolutional neural network (CNN), tailored for real-time solutions in the inverse statics of soft continuum robots within the Absolute Nodal Coordinate Formulation (ANCF) framework. The CNN model is adeptly designed to address the spatial continuities and inherent nonlinearity of ANCF, facilitating the resolution of complex inverse quasi-statics in soft continuum robotics. The model demonstrates an acceptable accuracy in providing ANCF solutions with minimal tolerance levels, effectively tackling challenging inverse quasi-static problems in continuum robots.

Integrating the Deep CNN with the ANCF, as newly proposed in this research, marks a considerable improvement over the current approaches. It offers a more powerful and efficient solution for statics modeling in soft continuum robotics. This advancement facilitates the resolution of inverse statics problems for soft robots modeled using ANCF. In our system, the model is designed to receive a specified target position for the end-effector and then compute the requisite external moments at the robot’s tip to achieve this position.

The study also underscores the utility of this approach in generating adequate trajectories for continuum robots by accurately determining the necessary moments to be applied at the robot’s extremities. Through comprehensive numerical studies, the efficacy of the proposed method has been established, highlighting its applicability in practical scenarios. However, further research is required to extend this method to the dynamic modeling of continuum robots using the ANCF framework. Future work should also explore the integration of CNN-based control in continuum robotics, along with the experimental validation of the deep learning models, in order to further substantiate and enhance the applicability of these methods.

In the evolving landscape of continuum robotics, the integration of deep learning models in data-driven model predictive control (MPC), especially when applied to robots modeled using the Absolute Nodal Coordinate Formulation (ANCF), presents a fertile ground for future research. The fusion of deep learning and MPC opens up innovative possibilities, ranging from enhanced learning algorithms capable of comprehending complex dynamics to the implementation of real-time control systems that can seamlessly integrate feedback and adjust to changing conditions. One of the paramount challenges in this domain is improving computational efficiency, which can be achieved through optimized neural network structures and the utilization of advanced hardware accelerators. This advancement is essential for ensuring the robustness and reliability of these systems across diverse operational environments, including industrial and medical applications.

## Figures and Tables

**Table 1:** Selected architectures for the five-fold cross validation experiment.

Model	Layers	Size	Average MSE
1	$F_{16},F_{3}$	259	4.19
2	$C_{8,2},F_{3}$	187	8.17
3	$C_{8,2},C_{4,2},F_{3}$	199	7.19
4	$C_{16,2},C_{8,2},F_{3}$	651	4.54
5	$C_{32,2},C_{16,2},F_{8},F_{3}$	2515	1.07

## Data Availability

Data is contained within the article.

## Abbreviations

The following abbreviations are used in this manuscript:

ANCF	Absolute Nodal Coordinate Formulation
CNN	Convolutional Neural Networks
MSE	Mean Square Error
CCM	Constant Curvature Model
